# Prevalence of Depression among Type 2 Diabetic Outpatients in Black Lion General Specialized Hospital, Addis Ababa, Ethiopia

**DOI:** 10.1155/2015/184902

**Published:** 2015-02-19

**Authors:** Tesfa Dejenie Habtewold, Yosef Tsige Radie, Nigussie Tadesse Sharew

**Affiliations:** ^1^Department of Nursing, College of Health Science, Debre Berhan University, Debre Berhan, Ethiopia; ^2^Department of Nursing and Midwifery, College of Health Science, Addis Ababa University, Addis Ababa, Ethiopia

## Abstract

*Background.* The emotional consequences of diabetes have been scrutinized by a number of investigative teams and there are varying reports about the association of depression with type 2 diabetes mellitus. However, there is limited data about this in Ethiopia. Therefore, the purpose of this study was to assess the prevalence of comorbid depression among type 2 diabetic outpatients. *Methods and Materials.* Institution based cross-sectional study design was conducted on a random sample of 276 type 2 diabetic outpatients from Black Lion General Specialized Hospital. Systematic random sampling technique was used to get these individual patients from 920 type 2 diabetic outpatients who have an appointment during the data collection period. Patients' depression status was measured using Patient Health Questionnaire 9 (PHQ 9). *Result.* Totally 264 type 2 diabetic outpatients were interviewed with a response rate of 95.6%. The prevalence of depression among type 2 diabetic outpatients was 13%. Based on PHQ 9 score, 28.4% (75) fulfilled the criteria for mild depression, 12.1% (32) for moderate depression, 2.7% (7) for moderately severe depression, and 1.5% (4) for severe depression. But 45.8% (121) of patients had no clinically significant depression. *Conclusion.* This study demonstrated that depression is a common comorbid health problem in type 2 diabetic outpatients with a prevalence rate of 13%.

## 1. Background

### 1.1. Introduction

Diabetes mellitus is a chronic metabolic disease, characterized by a disorder in the metabolism of carbohydrates, lipids, and amino acids, either as a result of decreased insulin secretion or due to a reduction to insulin sensitivity of the cells of the body cells. It is a disease that acquires epidemic form and constitutes one of the major threats to human health in the 21st century [1].

The World Health Organization projected that 300 million people will suffer from diabetes by 2025 [2]. Ethiopia, which is one of the developing nations, is at a risk of increased diabetes incidence. In 2012 the estimated diagnosed diabetes cases (20–79 years) and estimated numbers of people with undiagnosed diabetes (20–79 years) in 1000s in Ethiopia are 1,386.64 and 1,145.50, respectively. The estimated diabetes-related death is 23,869 (20–79 years) [3].

The emotional consequences of diabetes have been scrutinized by a number of investigative teams [4]. A growing body of literature has established a strong association between depression and type 2 diabetes [2, 4–7]. And the negative impact depression can have on quality of life together with the increased healthcare costs of comorbid depression for people with diabetes have been recognized [7]. Correspondingly surveys and meta-analyses conducted on diabetes mellitus and depression have shown that comorbid depression was common and the existence of diabetes mellitus doubles/triples the probabilities of depression occurrence [6–16]. Comorbid depression and diabetes may significantly worsen the course of both disorders, leading to increased socioeconomic stress, reduced functioning and quality of life, and higher complication and mortality rates [17]. On the contrary, O'Connor et al. compared diabetic and nondiabetic samples of patients in Great Britain and found no clear relationship between onset of diabetes and depression [18].

### 1.2. Prevalence of Comorbid Depression

The prevalence of depression has varied tremendously by definition, study design, source of subjects, study area, time frame, and measurement methods in previous studies. Thus, it is difficult to accurately estimate the potential medical care needs and public health burdens of depression in the general diabetic population [19]. A number of studies, including systematic review and meta-analyses, have shown the occurrence of depression among type 2 diabetic patients [20].

Studies from USA and UK reported the prevalence of depression in patients with type 2 diabetic patient varying from 30 to 83% [19, 21]. In addition, prevalence estimates among Hispanics indicate that diabetes is associated with a twofold higher risk of comorbid depression compared to the general population, with rates as high as 33% [7]. Similarly a quarterly publication and prevalence study in Mexico stated that 31% of people with type 2 diabetes report significant depressive symptoms and approximately 11% meet criteria for a major depressive disorder [14] and 46% of patients with Type 2 diabetes diagnosed using the Beck Depression Inventory [22], respectively. Acee also found that 31.1% of older Mexican American diabetics reported clinically significant levels of depressive symptoms [23].

In a study from the European Depression in Diabetes (EDID) research consortium, prevalence rates for depressive affect in outpatients with diabetes ranged between 34% and 39% for Croatian, 19% and 21% for Dutch, and 19% and 39% for English [24].

Other studies conducted in Asian countries also support the above findings from European and American countries. A cross-sectional study conducted in Chandigarh, India, discovered that, from 300 type 2 diabetic patients, 23% (68) fulfilled the criteria for severe depression, 18% (54) for moderate depression, and 59% (178) had no clinically significant depression [2]. Likewise, a cross-sectional study done in Bangladesh using PHQ-9 (score ≥5) depicted that the prevalence of depressive symptoms was 34% (142). When a cut-off value (PHQ-9 ≥10) indicative of moderate to severe depression was used, the prevalence was to be 16.5% (69) which was supportive of the above findings [25].

Besides there are studies, systematic reviews, and meta-analysis which reported similar result. A study done in South Australia [20] and Iran [26] found that the prevalence of depression in patients with type 2 diabetes mellitus was 23.6% and 55%, respectively. Another study showed that the rates of elevated depressive symptoms have been found to be 27% in type 2 diabetes mellitus [8].

Furthermore, several reviews indicate that the prevalence of comorbid major depressive disorder (MDD) in persons with diabetes ranges from 11 to 33% [13]. Acee reviewed 20 studies of the comorbidity of depression and diabetes and found that the prevalence of current depression in diabetic samples averaged around 15%, much higher than in the general population [23].

In the same way, a meta-analysis by O'Connor et al. identified the prevalence of depression in diabetes ranging from 8 to 61% [18].

Similar research from Africa also reported comparable finding. A cross-sectional study at Jos University Teaching Hospital, Jos, Nigeria discovered that the prevalence rate of depression among diabetic outpatients was 19.4% [27].

In Ethiopia mental health has been one of the most disadvantaged health programs, both in terms of facilities and trained manpower. However, during the last decade, encouraging efforts have been taken to expand services throughout the country [28].

However, in spite of the huge impact of comorbid depression and diabetes on the individual and its importance as a public health problem, little is known about the magnitude of depression in people with diabetes in Ethiopia.

## 2. Significance of the Study

Clinical guidelines advise screening for depression in patients with diabetes [5]. A timely identification of patients with subthreshold or clinical depression and a structured approach for the management of depression in diabetes has proved to be effective in reducing the burden of depression in diabetes. In the short term, healthcare expenditure can be saved. In the long term, a better prognosis, maintenance, or improvement in quality of life can be achieved in patients with diabetes, which is the ultimate goal of diabetes therapy [29]. Previous research into the prevalence of depression in diabetes has enhanced our understanding of the magnitude of the problem of depression and its ramifications. But only a few studies have been conducted in specialist diabetes care settings.

Data on depression among diabetes patients in Ethiopia are inadequate particularly for type 2 diabetic outpatients. Therefore, this study will be helpful in extending understanding of the relationship of depression to diabetes thoroughly investigating prevalence of depression in type 2 diabetic outpatients. Additionally, it will help health care providers to initiate early diagnosis and management of depression based on a research finding, and provides policy makers and NGOs with relevant information for future planning and interventions. At last, it will be used as input for further research.

## 3. Objective

To assess the prevalence of comorbid depression among type 2 diabetic outpatients presenting to Black Lion General Specialized Hospital, Addis Ababa.

## 4. Methods and Materials

### 4.1. Study Area

Black Lion (Tikur Anbessa in Amharic) General Specialized Hospital, located in the nation's capital Addis Ababa, is Ethiopia's largest general public hospital and one of University Hospitals in the country. The hospital provides a tertiary level referral treatment and is open for 24 hours for emergency services. Black Lion General Specialized Hospital offers diagnosis and treatment for approximately 370,000–400,000 patients a year [30].

There are different units and clinics that provide specialized service for clients and patients. Among these clinics diabetes clinic was one of the clinics inaugurated by Prof. Dr Giuseppe “Pino” Grimaldi (president of the international association of lions clubs) on Saturday 12th November 1994. In diabetes clinic approximately 115 type 2 diabetic patients were seen weekly.

### 4.2. Study Design

Institution based cross-sectional study design was used to assess the prevalence of comorbid depression among patients with type 2 diabetes mellitus.

### 4.3. Study Period

The study was conducted from September 23, 2013, to May 13, 2013.

### 4.4. Source Population

All type 2 diabetes outpatients are on follow-up treatment in diabetes clinic.

### 4.5. Study Population

Type 2 diabetes outpatients have follow-up appointment during data collection period.

### 4.6. Sample Size Determination

The actual sample size for the study was determined using single population proportion formula:

(1) ni=Za/22pqd2,

where *n*_*i*_ = required initial sample size, *Z*_*a*/2_ = critical value for normal distribution at 95% confidence interval which equals 1.96 (*Z* value at alpha = 0.05), *P* = proportion of success; that is, the prevalence of depressive symptoms using the PHQ-9 (score ≥ 5) was 34% [25], *q* = proportion of type 2 diabetic population not having comorbid depression (0.66), and *d* = marginal error (0.05):

(2) $$\begin{array}{c} n_{i}=\frac{{\left(1.96\right)}^{2}\times 0.34\times 0.66}{{\left(0.05\right)}^{2}} \end{array}$$

*n*_*i*_ = 345.

Since the sampling was made from finite population (*N* < 10, 000), it needs finite population correction. Therefore,

(3) $$\begin{array}{c} n_{f}=\frac{n_{i}}{1+n_{i}/N},\\ n_{f}=\frac{345}{1+345/920}, \end{array}$$

where *n*_*f*_ was the final sample size, *n*_*i*_ was the initial sample size determined using the formula, and *N* was the size of the source population. By considering 10% nonresponse rate, the total sample size was 276 type 2 diabetic outpatients.

### 4.7. Sampling Procedure

**Figure 1:**
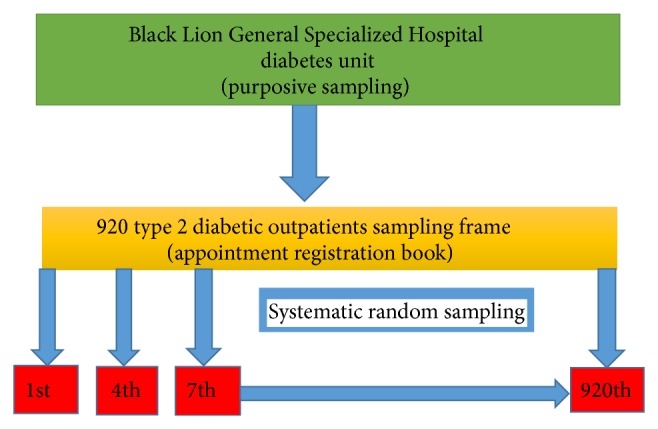
Schematic presentation of sampling procedure for the study in Black Lion General Specialized Hospital, Addis Ababa, Ethiopia, 2013.

As illustrated in Figure 1, these 276 samples were selected by using systematic random sampling technique. The individual type 2 diabetic outpatient was approached through calculating sampling interval *K* (*N*/*n*, where *N* is the total number of type 2 diabetic patients who have appointment during the data collection period which was approximately 920 and *n* was the calculated final sample size which was 276):

 
*K* = 920/276;
 
*K* = 3.3 ≈ 3.

So that the individual type 2 diabetic outpatient was interviewed every *K*th; that is, every 3rd patient was selected from sampling frame developed by giving a number for all 920 patients in the registration book ascendingly. Since the sampling interval was 3, a number between 1 and 3 was selected randomly by lottery method and number 1 was drawn first to take as a starting patient for the interview.

### 4.8. Inclusion and Exclusion Criteria

#### 4.8.1. Inclusion Criteria

Patients were approached by the research team and invited to participate in the study that were as follows:

- diagnosed as type 2 diabetic patient based on laboratory result (blood sugar level) and clinical finding for at least one year,
- age ≥ 20 years old,
- being capable of independent communication and giving informed verbal consent.

#### 4.8.2. Exclusion Criteria

The exclusion criteria are as follows:

- Patients with type 1 diabetes mellitus.
- Patients who were currently being treated for depression,
- age <20 years old.
- Not capable of independent communication.
- Patients who have refused to participate in the study.

### 4.9. Method of Data Collection

Quantitative and qualitative data was collected by using structured interviewer administered questionnaire. Demographic and health related information was collected from each patient and medical records using data abstraction form. Depression status of patients was ascertained at the time of recruitment. The Patient Health Questionnaire 9 (PHQ 9) was used to evaluate depression status of patients.

Patients with established type 2 diabetes mellitus were evaluated for depression by administering nine-item Patient Health Questionnaire 9 (PHQ 9) adapted from Pfizer Inc. using local language.

### 4.10. Variable Specification

#### 4.10.1. Dependent Variables

Dependent variable is the response that is measured and it is the presumed effect. In this research the dependent variable is;

- Depression.

#### 4.10.2. Independent Variables

Independent variable is the variable that is varied or manipulated by the researcher, and the presumed cause. In this research the independent variable is;

- Type 2 diabetes mellitus.

### 4.11. Operational Definition

- No depression: Patient Health Questionnaire 9 score is 0.
- Minimal depression: Patient Health Questionnaire 9 score is 1–4.
- Mild depression: Patient Health Questionnaire 9 score is 5–9.
- Moderate depression: Patient Health Questionnaire 9 score is 10–14.
- Moderately severe depression: Patient Health Questionnaire 9 score is 15–19.
- Severe depression: Patient Health Questionnaire 9 score is 20–27.

### 4.12. Data Processing and Analysis

After checking collected data visually for completeness, the response was coded and entered into the computer using EPI info version 3.5.1. Statistical packages, and then 10% of the responses was randomly selected and checked for the consistency of data entry. Then printed frequencies were used for checking of outliers and for cleaning data. Data was cleaned accordingly and then exported to SPSS version 20.0 (IBM SPSS Corp.) for further analysis. The frequency distribution of dependent and independent variables were worked out and presented in table, figure, and graph.

### 4.13. Data Quality Issues

To assure quality of the data, properly designed data collection tool was prepared and pretested and training was given to data collectors. Additionally, on each data collection day, the collected data was reviewed and checked for its completeness by principal investigator and appropriate design and sampling procedure was applied. Moreover, the exclusion criteria were considered.

### 4.14. Ethical Considerations

In order to follow the ethical and legal standards of scientific investigation, the study was conducted after approval of the proposal by Addis Ababa University institutional review board. Participation was voluntary and information was collected anonymously after obtaining verbal consent from each respondent by assuring confidentiality throughout the data collection period.

## 5. Result

### 5.1. Sociodemographic Characteristics

Totally 264 type 2 diabetic outpatients were interviewed with a response rate of 95.6%. As shown in Table 1, of whom those interviewed patients 53.0% (140) were female, 69.3% (183) were married, 80.7% (213) were Orthodox Christian, and 57.2% (151) were Amhara. In addition, the mean ± SD age at diagnosis and current age of patients were 43.9 ± 10.9 and 55.9 ± 10.9 years, respectively. Besides, 86.4% (228) lived in Addis Ababa, and 61.7% (163) were with waist circumference of ≥ 95cm (mean ± SD, 98.9 ± 11.1). Likewise, the median monthly income of the family was 750 ETB (651-1400 ETB) and 33.7% (89) were attended college/university level education.

### 5.2. Clinical Characteristics

As illustrated in Table 2, 43.2% (145) of patients were on oral hypoglycemic treatment, 78.3% (141) were with cardiovascular diseases (hypertension and heart failure), and 69.7% (140) were with diabetic retinopathy. Above and beyond, 58.7% (155) of patients reported 1 to 2 comorbid disease (mean ± SD, 1.1 ± 0.9) that is, evidenced by review of patient's medical record. Similarly, 50% (132) were with BMI ≤ 24.9 kg/m^2^ (mean ± SD, 25.4 ± 3.7) and reported physical disability. One hundred one (38.3%) patients were living with diabetes mellitus for ≤8 years and 25.0% (66) taking physician prescribed medication for >17 years (mean ± SD, 12 ± 7.9). Regarding the laboratory reported fasting blood glucose level, 12.9% (34) were with ≤100 mg/dL, 19.7% (52) were with 101–126 mg/dL, and 67.4% (178) were with ≥127 mg/dL.

### 5.3. Psychosocial Attributes

**Figure 2:**
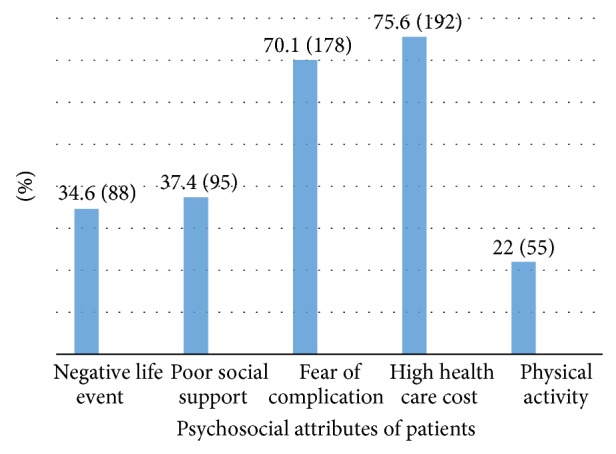
Distribution of psychosocial attributes among type 2 diabetic outpatients in Black Lion General Specialized Hospital, Addis Ababa, Ethiopia, 2013.

As depicted in Figure 2, 75.6% (192) were reported that health care cost for type 2 diabetes treatment was high and 22% (55) of patients do physical activity as recommended by the physician.

### 5.4. Reliability and Item Analysis

Cronbach's *α* for the PHQ-9 scale was 0.72 indicating acceptable consistency of this psychometric scale for the study population. The correlations between nine items of the PHQ-9 and total PHQ-9 scores ranged from 0.22 to 0.69, and all correlations were significant at the 0.01 level.

### 5.5. Prevalence of Depression

**Figure 3:**
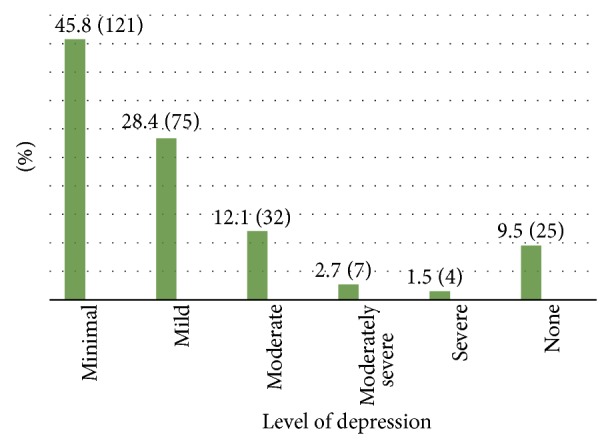
The prevalence of comorbid depression based on PHQ 9 score among type 2 diabetic outpatients in Black Lion General Specialized Hospital, Addis Ababa, Ethiopia, 2013.

**Figure 4:**
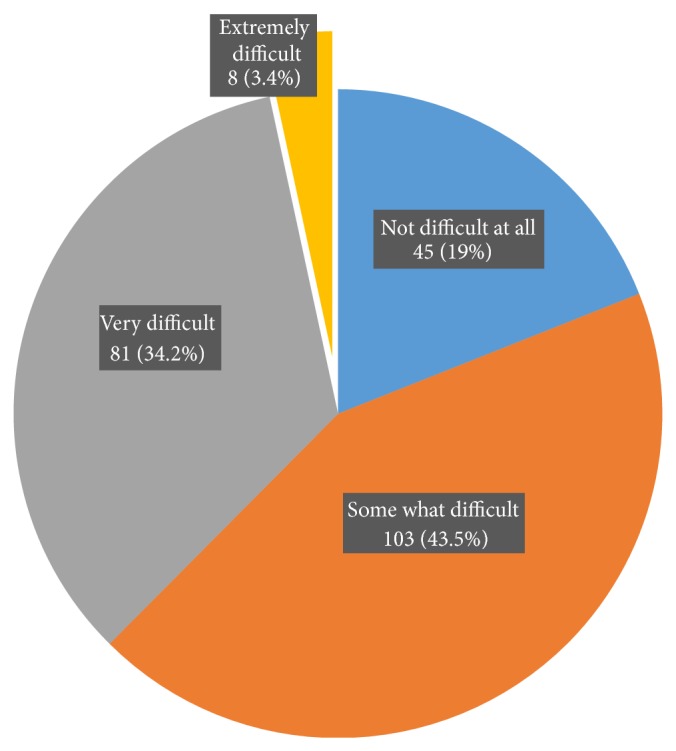
Level of difficulty of depressive symptoms among type 2 diabetic outpatients to do their work, take care of things at home, or get along with other people in Black Lion General Specialized Hospital, Addis Ababa, Ethiopia, 2013.

The mean ± SD of PHQ 9 score was 5.2 ± 4.6. Twenty five (9.5%) type 2 diabetic outpatients did not report any of depressive symptoms. As showed in Figure 3, from the rest of 239 type 2 diabetic outpatients, 28.4% (75) fulfilled the criteria for mild depression, that is, PHQ 9 score 5–9, 12.1% (32) for moderate depression, that is, PHQ 9 score 10–14, 2.7% (7) for moderately severe depression, that is, PHQ 9 score 15–19, and 1.5% (4) for severe depression, that is, PHQ 9 score 20–27. But, 45.8% (121) of patients had no clinically significant depression. Since only 118 patients reported clinically significant depression, the overall prevalence rate of depression among type 2 diabetic outpatients was found 13%. As depicted in Figure 4, 43.5% (103) of patients express their feeling that the presence of depressive symptoms made “somewhat difficult” to do work, take care of things at home, or to get along with other people.

## 6. Discussion

It is difficult to accurately estimate the potential medical care needs and public health burdens of depression in the general diabetic population [23]. However, in spite of the huge impact of comorbid depression and diabetes on the individual and its importance as a public health problem, little is known about the existence of depression in people with diabetes in Ethiopia. This study has tried to address this issue.

The purpose of this study was to assess prevalence of comorbid depression in a random sample of outpatients with type 2 diabetes mellitus in Black Lion General Specialized Hospital, Addis Ababa, Ethiopia. To our knowledge, this study was the first that has investigated depression among type 2 diabetic outpatients using a PHQ 9 psychometric scale.

This study revealed that from 264 type 2 diabetic outpatients, 28.4% (75) fulfilled the criteria for mild depression, that is, PHQ 9 score 5–9, 12.1% (32) for moderate depression, that is, PHQ 9 score 10–14, 2.7% (7) for moderately severe depression, that is, PHQ 9 score 15–19, and 1.5% (4) for severe depression, that is, PHQ 9 score 20–27. But, 45.8% (121) of patients had no clinically significant depression and 9.5% (25) of patients did not report any of depressive symptoms. When a cut-off score of PHQ 9 ≥ 10 (i.e. moderate to severe depression) was used, the prevalence was 18% (43). However, the prevalence of depression was 49.4% (118) when a cut-off score of PHQ 9 ≥ 5 was used.

This finding was comparable with the result from a cross-sectional study done in Bangladesh using PHQ 9 psychometric tool, depicted that the prevalence of depressive symptoms using PHQ-9 (score ≥ 5) was 34% (142). When a cut-off value (PHQ-9 ≥ 10) indicative of moderate to severe depression was used, the prevalence was to be 16.5% (69) [25].

On the other hand this finding was relatively lower than a cross-sectional study conducted in Chandigarh, India; discovered that from 300 type 2 diabetic patients, 23% (68) fulfilled the criteria for severe depression, 18% (54) for moderate depression and 59% (178) had no clinically significant depression [2]. This discrepancy might be due to variation in sample size.

From the total of 920 type 2 diabetic outpatient candidates, only 118 patients reported clinically significant depression. Therefore, the overall prevalence rate of depression among type 2 diabetic outpatients was found 13%. This finding was smaller than the finding of a cross-sectional study at Jos University Teaching Hospital, Jos, Nigeria that discovered the prevalence rate of depression among diabetic outpatients was 19.4% [27], a small study from Iran that reported 55% prevalence of depression in patients with type 2 diabetic patients [26] and a study done in Mexico, the prevalence of depression, diagnosed using the Beck Depression Inventory, was found to be 46% in patients with Type 2 diabetes [15, 22]. Correspondingly, it was lower by half from the review report by McIntyre et al. who showed that the prevalence of depressive symptoms has been found to be 27% in type 2 diabetes mellitus [8].

These discrepancies of prevalence might be due to variation in attributes of study participants, use of different psychometric scale, study design, setting, time frame, and the level of country development.

Providing the patients with the results of blood sugar, cholesterol, blood pressure, and medications plan through outpatient service is not enough itself to improve service delivery and bring about change [25]. The findings of this study have major implications for clinical practice in the Black Lion General Specialized Hospital and other health care settings in the country, where clinicians' recognition of mental disorder rates is low and improving recognition rates is a challenge because of high patient loads and poor undergraduate training in these skills.

## 7. Strength and Limitation

The strengths of this study include a high response rate and the inclusive nature of this research as individuals could participate regardless of literacy level. Additionally, rather than having to rely on self-report, health related information was collected from patients' medical records. Also, a reasonable sample size and ascertaining depression with culturally standardized questionnaires are strengths of this study. At last, since it was the first study in type, it will provide basic information for those who are interested.

However, an important limitation of this study was that Patient Health Questionnaire 9 psychiatric scale was used which was not the gold standard method to assess depression. Likewise there was absence of similar study done in Ethiopia health care setting to compare the finding during discussion.

## 8. Conclusion

In conclusion, this study demonstrated that depression is a common comorbid health problem in type 2 diabetic outpatients in Black Lion General Specialized Hospital, Ethiopia. Within this sample of outpatients with type 2 diabetes mellitus, the study found that the prevalence rate of depression was 13%.

This study provides rich data on the prevalence of depression in type 2 diabetic outpatients in Ethiopia. In a setting where recognition, screening, and treatment levels remain low, health care providers need to focus their efforts on diagnosing, referring and effectively treating comorbid depression in order to deliver rights-based and client-centered services for people in real needs.

## Figures and Tables

**Table 1:** Sociodemographic characteristics of type 2 diabetic outpatients in Black Lion General Specialized Hospital, Addis Ababa, Ethiopia, 2013. (*n* = 264).

Variable	Category	Frequency (*n*)	Percent (%)
Sex	Male	124	47.0
	Female	140	53.0

Residence	Addis Ababa	228	86.4
	Outside Addis Ababa	36	13.6

Current age	<=49	60	22.7
	50–54	53	20.1
	55–59	48	18.2
	60–64	43	16.3
	>=65	60	22.7

Age at diagnosis	<=34	49	18.6
	35–39	45	17.0
	40–44	43	16.3
	45–49	49	18.6
	50–54	27	10.2
	>=55	51	19.3

Marital status	Single	9	3.4
	Married	183	69.3
	Divorced	24	9.1
	Widowed	48	18.2

Religion	Orthodox	213	80.7
	Muslim	24	9.1
	Protestant	20	7.6
	Catholic	2	0.8
	Others	5	1.9

Ethnicity	Amhara	151	57.2
	Oromo	40	15.2
	Tigre	25	9.5
	Gurage	29	11.0
	Others	19	7.2

Educational status	Cannot read and write	35	13.3
	Read and write only	16	6.1
	Primary school (1–8)	65	24.6
	Secondary (9–12)	59	22.3
	College/University	89	33.7

Occupation	Farmer	6	2.3
	Civil servant	47	17.8
	Merchant	10	3.8
	House wife	47	17.8
	Private worker	38	14.4
	Pensioned	58	22.0
	No employment	48	18.2
	Others	10	3.8

Monthly family income (ETB)	<=650	125	47.3
	651–1400	61	23.1
	>=1401	78	29.5

Waist circumference (cm)	<95	101	38.3
	>=95	163	61.7

**Table 2:** Clinical characteristics of type 2 diabetic outpatients in Black Lion General Specialized Hospital, Addis Ababa, Ethiopia, 2013. (*n* = 264).

Variable	Category	Frequency	Percent (%)
Diabetes treatment regimen	Single insulin	108	40.9
	Combined insulin	12	4.5
	Insulin plus oral hypoglycemic	30	11.4
	Oral hypoglycemic	114	43.2

Duration of diabetes (years)	<=8	101	38.3
	9–16	96	36.4
	17+	67	25.4

Duration of diabetes treatment (years)	<=8	105	39.8
	9–16	93	35.2
	17+	66	25.0

Comorbid disease^a^	Cardiovascular disease	141	78.3%
	Respiratory disease	17	9.4%
	Renal disease	13	7.2%
	Neurologic disease	4	2.2%
	Other comorbid disease	80	44.4%

Complication of diabetes^b^	Diabetic retinopathy	140	69.7%
	Diabetic nephropathy	69	34.3%
	Diabetic neuropathy	83	41.3%
	Sexual dysfunction	69	34.3%

Body mass index	<=24.9	132	50.0
	25.0–29.9	98	37.1
	>=30	34	12.9

Fasting blood glucose	<=100	34	12.9
	101–126	52	19.7
	>=127	178	67.4

Number of comorbidities	0	83	31.4
	1-2	155	58.7
	>=3	26	9.8

Number of prescribed medication administrations per day	<=4	77	29.2
	5-6	113	42.8
	7+	74	28.0

Number of diabetic complications	0	65	24.6
	1-2	148	56.1
	>=3	51	19.3

Physical disability	Yes	132	50.0
	No	132	50.0

Medication burden	≤3	77	29.2
	≥4	187	70.8

a: number of respondents is 180 and b: number of respondents is 201.

## List of Acronyms and Abbreviations

BMI: Body mass index
CAD: Coronary artery diseases
CI: Confidence interval
DSM: Diagnostic statistical manual of mental disorders
EDID: European depression in diabetes
E.g.: Example
et al.: Et alia
ETB: Ethiopian birr
FBS: Fasting blood sugar
FDRE: Federal Democratic Republic of Ethiopia
FMOH: Federal ministry of health
IBM: International business machine
Kg/m^2^: Kilogram per meter square
MDD: Major depressive disorder
MOH: Ministry of Health
NGOs: Nongovernmental Organizations
PHQ: Patient Health Questionnaire
SD: Standard deviation
SPSS: Statistical package for social science
T2DM: Type 2 diabetes mellitus
UK: United Kingdom
USA: United States of America
Vs.: Versus.
